# The Move Prover

**DOI:** 10.1007/978-3-030-53288-8_7

**Published:** 2020-06-13

**Authors:** Jingyi Emma Zhong, Kevin Cheang, Shaz Qadeer, Wolfgang Grieskamp, Sam Blackshear, Junkil Park, Yoni Zohar, Clark Barrett, David L. Dill

**Affiliations:** 8 Microsoft Research Lab, Redmond, WA USA; 9 University of Southern California, Los Angeles, CA USA; 10 Stanford University, Stanford, USA; 11 UC Berkeley, Berkeley, USA; 12 Novi, Seattle, WA USA; 13 Novi, Menlo Park, CA USA

**Keywords:** Libra, Blockchain, Smart contracts, Formal verification

## Abstract

The Libra blockchain is designed to store billions of dollars in assets, so the security of code that executes transactions is important. The Libra blockchain has a new language for implementing transactions, called “Move.” This paper describes the Move Prover, an automatic formal verification system for Move. We overview the unique features of the Move language and then describe the architecture of the Prover, including the language for formal specification and the translation to the Boogie intermediate verification language .

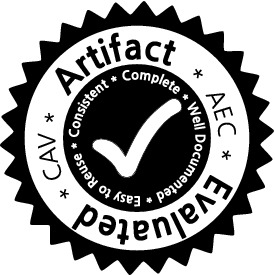



## Introduction

The ability to implement arbitrary transactions on a blockchain via so-called *smart contracts* has led to an explosion in innovative services in systems such as Ethereum [41]. Unfortunately, bugs in smart contracts have led to massive amounts of funds being stolen or made inaccessible [5, 15]. In retrospect, the source of these disasters is fairly obvious: smart contracts operate without a safety net. A fundamental requirement for blockchains is that transactions be automatic and irreversible. Unlike traditional financial applications, there is little opportunity for humans to oversee or intervene in transactions. Indeed, the design of the blockchain is intended to prevent human involvement. The resulting potential havoc that can be caused by a bug in a smart contract makes it essential for these contracts to be correct, without vulnerabilities. Not surprisingly, there is great interest in formal verification and other advanced testing methods for smart contracts, and several verification systems already exist or are under development.

The Libra blockchain [3, 38] is designed to be a foundation for supporting financial services for billions of people around the world. If successful, it could store and manage assets worth billions of dollars, with correspondingly stringent security requirements. The code that modifies the state of the blockchain is especially important. The architecture of the Libra blockchain requires that all such modifications be performed by the Move [12] virtual machine, which executes the well-defined Move instruction set. This architecture means that verification efforts can focus on the correctness of bytecode programs implementing smart contracts, including formally verifying those programs.

**Contributions**

In this paper, we describe a specification language and formal verification system for Move. If a programmer writes functional correctness properties for a procedure, the Move Prover tool can automatically verify it. Although many similar Floyd-Hoare verifiers exist, widespread adoption has been a challenge because conventional software is large, complex, and uses language features that present difficulties for even the simplest verification tasks. However, we are hopeful that the Move Prover will be used by the majority of Move programmers. There are three reasons for this optimism. First, the Move language has been designed to support verification. Second, we are building a culture of specification from the beginning: each Move module used by the Libra blockchain is being written with an accompanying formal specification. Finally, we are working to make the Move Prover as precise, fast, and user-friendly as possible.

The Move language, the Move Prover, Move programs, and their specifications, have been evolving rapidly, so this description necessarily represents a snapshot of the project at a particular time. However, we expect most of the changes to be improvements and extensions to the basics described here. In the remainder of this paper, we will:

1. Present a brief overview of Move and explain the language design decisions that facilitate verification (Sect. 2);
2. Describe how the Move Prover toolchain is implemented (Sect. 3);
3. Explain the model used to represent Move programs (Sect. 4);
4. Define the Move specification language and give examples of useful properties it can encode (Sect. 5); and
5. Demonstrate that the Move Prover can verify important aspects of the Libra core modules (Sect. 6).

## Background: The Move Language

Move [12] is an executable bytecode language for writing smart contracts and custom transaction logic. Contracts in Move are written as *modules* that contain record types and procedures. Records in modules may either be struct or *resource* types—the most novel feature of Move. A resource type has linear [17] semantics, meaning that resources cannot be created, copied, or destroyed except by procedures in its declaring module. Resources allow programmers to encode safe, yet customizable assets that cannot be accidentally (or intentionally) copied or destroyed by code outside the module.

Move is minimal in comparison to most conventional programming languages. The only types besides records are primitives (Booleans, unsigned integers, addresses), vectors, and references (which must be labeled as mutable or immutable, similar to Rust [30]). Records can contain primitives and other records, but not references. Control-flow constructs can be encoded via jumps to static labels in the bytecode.

Move programs execute in the context of a blockchain with modules and resources published under *account addresses*. To interact with the blockchain, a programmer can write a Move *transaction script*, a single-procedure program similar to a  procedure in a conventional language, that invokes procedures of published modules. This script is then packaged into a cryptographically signed transaction that is executed by validators in the Libra blockchain. As in Ethereum, transaction execution is *metered*, meaning that computational resources (or “gas”) used when a Move program is executed are measured and must be paid for by the submitter of a transaction (though we note that the Move Prover does not yet reason about gas usage).

**Fig. 1.**
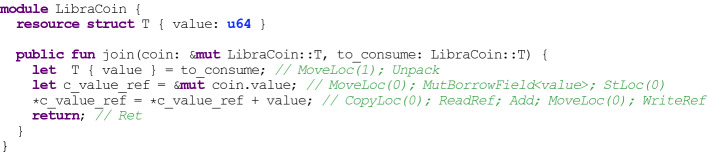
A Move module with its bytecode representation in comments.

*Verification-Friendly Design.* There are several aspects of Move ’s design that facilitate verification. The first is limited interaction with the environment: to ensure deterministic execution, the language can only read data from the global blockchain state or the current transaction (no file or network I/O). Second, many features that are challenging for verification are absent from Move: concurrency, higher-order functions, exceptions, sub-typing, and dynamic dispatch. The absence of the last feature is particularly notable because it is present in Ethereum bytecode and has contributed to subtle *re-entrancy* bugs (e.g., [14]). Third, Move has built-in safe arithmetic: overflows and underflows are detected during execution and result in a transaction abort. Finally, many common errors are prevented by the Move *bytecode verifier* (not to be confused with the Move Prover), a static analyzer that checks each bytecode program before execution (similar to the JVM [26] or CLR [31] bytecode verifier). The bytecode verifier ensures that:

1. Procedures and struct declarations are well-typed (e.g., linearity of resources)
2. Dependent modules and procedure targets exist (i.e., static linking)
3. Module dependencies are acyclic
4. The operand stack height is the same at the beginning and end of each basic block
5. A procedure can only touch stack locations belonging to callers via a reference passed to the callee
6. The global and local memory are always tree-shaped
7. There are no dangling references
8. A mutable reference has exclusive access to its referent

Because these checks are run on every Move bytecode program, the prover can rely on them in its own reasoning. Note that this would not be true if the checks were performed by a source language compiler, since bad bytecode programs could be created by compiler bugs or by writing programs directly in the executable bytecode representation.

**Fig. 2.**
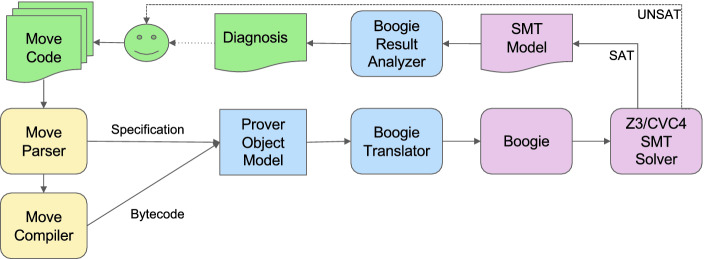
The Move Prover architecture.

*Limited Aliasing.* In the rest of this section, we present an example that explains the memory-related invariants enforced by the Move bytecode verifier (6–8 above). The example in Fig. 1 is written in the Move source language, which can be directly compiled to the Move bytecode representation shown in the comments (note that the Move Prover analyzes the bytecode itself). The  procedure accepts two arguments:  of type  (a mutable reference to a  value stored elsewhere) and  of type  (an *owned*  value). The purpose of this procedure is to destroy the  resource stored in  and add its value to the  resource referenced by  . The first line of the procedure performs the destruction by “unpacking”  (placing the program value bound to its field into the program variable  ), and the next two lines read the current value of  and update it.

The careful reader might wonder: what will happen if  is a reference to  ? In a C-like language, the first line would make  into a dangling reference, which would lead to a memory error when it is subsequently used. Fortunately, the Move bytecode verifier ensures that this cannot happen. An owned value like  can only be moved (either onto the operand stack or into global storage) if there are no outstanding references to the value. In addition, the bytecode verifier guarantees that no mutable reference can be an ancestor or descendant of another (mutable or immutable) reference in the same local or global data tree. This is a very strong restriction! It ensures that procedure formals that can be mutated (mutable references or owned values) point to disjoint memory locations. For example, an additional formal of type  in the code above could not point into the memory of the other formals. Formals that are immutable references may alias with each other, but not with mutable references or owned values. This means it is impossible for an update to a reference to affect the value retrieved by a simultaneously existing reference. These restrictions on the structure of memory enable greatly simplified reasoning about aliased mutable data, a significant challenge for verification in conventional languages.

## Tool Overview

Figure 2 shows the architecture of the Move Prover. The prover takes as input Move source code annotated with specifications. The overall workflow consists of several steps. First, the specifications are extracted from the annotated code, and the Move source code is compiled into Move bytecode. Next, all stack operations are removed from the bytecode and replaced with operations on local variables, and the stackless bytecode is abstracted into a prover object model. Along a separate path, the specifications are parsed and added to the prover object model. The finalized model is translated to a program in the Boogie *intermediate verification language (IVL)* [23, 24].

The Boogie program is handed to the Boogie verification system, which generates an SMT formula in the SMT-LIB format [10]. This can then be checked using an SMT solver such as Z3 [32] or CVC4 [9]. If the result of this check is UNSAT, then the specification holds, which is reported to the user. Otherwise, a countermodel is obtained from the SMT-solver, which gets translated back to Boogie. Boogie produces a Boogie-level error report, and this result is analyzed and transformed into a source-level diagnosis that is given back to the user. Using this diagnosis, the user can refine the implementation and/or specification and start the process again.

The prover is written in Rust and can be found in the   directory in the Libra repository on GitHub [25].^1^ We describe the Boogie model and the specification language in more detail in the following sections.

## Boogie Model

Boogie IVL is a simple imperative programming language that supports local and global variables, branching and loops, and procedures and procedure calls. Boogie is designed for verification, so it also supports pre- and post-conditions, loop invariants, and global axioms. Boogie programs are not executable; instead, they are provided as input to the Boogie verification system, which applies a verification strategy to generate verification conditions (as SMT formulas) [8]. If all of the verification conditions hold, then each procedure ensures its post-conditions, under the assumption that its pre-conditions hold. The variable types supported by Boogie IVL match the sorts supported by SMT solvers, e.g., Booleans, integers, arrays, bitvectors, and datatypes. This makes the translation of Boogie verification conditions into SMT formulas fairly transparent. Boogie is used as a back-end for a wide variety of verification tools. The general strategy is to model the semantics of a source language in Boogie. Then, programs and specifications in the source language can be translated into Boogie IVL and checked using the Boogie verification system. For more details about Boogie, we refer the reader to [1, 7, 23, 24].

**Fig. 3.**
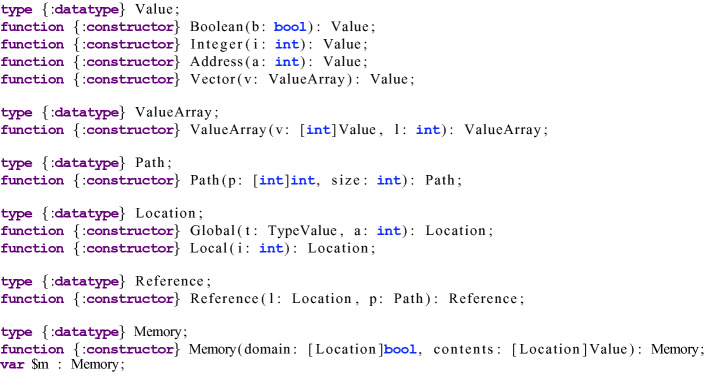
Highlights of the Boogie model for the Move Prover. The  syntax is used to declare a new datatype, and the  syntax is used to declare datatype constructors with their selectors. An array indexed by type  containing elements of type  is denoted in Boogie as  .

Following this pattern, we built a Boogie model for Move bytecode programs. A few highlights of the model are shown in Fig. 3 and described below. For a detailed understanding of the model, we refer the reader to the full Boogie model, which can be found in the Libra repository at   and to a formalization of the core Move bytecode language described in [13].

As mentioned above, in Move, a data value is either a primitive value (e.g., Boolean, integer, address), a struct (i.e. a record) containing one or more data values, or a vector of data values. Data values are represented in Boogie as the  datatype, with one constructor for each primitive type, plus a *vector* constructor (containing one field: a finite array of  ), used to model both vectors and structs.

Because Move supports generic functions (i.e. type-parameterized functions), we define a similar Boogie datatype for types called  (not shown). A type-parameterized function can then be represented as a Boogie procedure whose initial arguments are of type  (for the type parameters) and whose data arguments are of type  (regardless of their actual Move type). The bytecode verifier ensures type-correctness, so we do not check that types are used correctly, but rather assume this is the case (by using Boogie  statements as needed).

The  and  datatypes are mutually recursive, and thus a  can be thought of as a finite tree. A primitive  is a leaf node of the tree, while a struct or vector  is an internal node. A position within the tree can be uniquely identified by a *path*, which is a sequence of integers. A path specifies a node of the tree by starting at the root node and then following children according to the indices in the path. We model paths as finite arrays (also shown in Fig. 3). This simplifies the specification that two trees are disjoint, which is a necessary precondition in some smart contract functions.

A  can be stored in either local or global state, and references to data in either are allowed as local variables. For simplicity and uniformity, we have a single memory object which is a map from  to  (because memory is a partial function, it also contains a map from  to  , which indicates whether a particular location is present in memory). A  is either global (indexed by an account address and a type) or local (indexed by an integer). References are then represented as a pair consisting of a location and a path. To model reading from or writing to a reference, the global memory is accessed along the reference’s path. Note that this is done by enumerating cases up to the maximum possible path depth (based on the data structures in the modules being verified).^2^

Finally, each bytecode instruction is modeled as a procedure modifying local or global state in Boogie. A bytecode program is then translated to a sequence of procedure calls, with  statements handling control-flow.

## Specifications

**Fig. 4.**
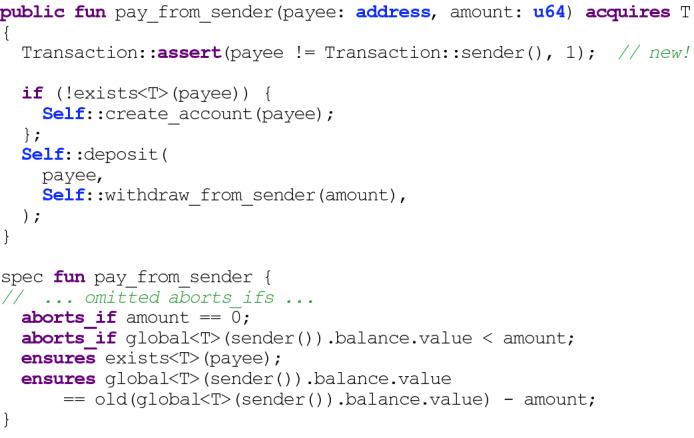
A simplified version of an example where verification led to an insight about a function. Without the  marked “new,” the specification fails to hold if  and  are the same, as explained in Sect. 6.

The Move Prover has a basic specification language for individual functions. Specifications include classical Floyd-Hoare pre-conditions, post-conditions, and a new condition specifying when a function aborts. (We are expanding this functionality to include ghost variables and global invariants for modules.) These conditions are separated from the actual code, in “spec blocks,” which are linked by name to the structure or function being specified, or to the containing module. Specifications never affect the execution of a module. A simplified example based on verifying a real Libra module appears in Fig. 4.

Pre-conditions and post-conditions are standard. Pre-conditions are introduced by the reserved word  and post-conditions are introduced by  , and each is followed by a Boolean expression, in a syntax that is very similar to Move, which includes the usual relational and arithmetic operators, record field access, etc. A sub-expression after  can be enclosed in  , causing the expression to be evaluated using the variable values in the program state immediately after entry to the function, instead of using the program state just before exit from the function. Move functions can return multiple values, so the expressions  ,  , etc. represent those return values.

Formal verifiers for conventional programming languages treat run-time errors as bugs to be reported. However, as in most smart contract languages, performing an undefined operation in Move, such as division by zero, cancels the entire transaction with no effect on the state except the consumption of some currency to pay for the computational resources consumed by the code that was executed before the error occurred. In Libra, this event is called an *abort*. Aborts are not necessarily run-time errors in Move. They are the standard way to handle illegal transactions, such as trying to perform an operation that is not authorized by the sender of the transaction.

Instead of treating all possible abort conditions as bugs, the Move Prover allows the user to specify the conditions under which a function is expected to abort. This type of specification is introduced by the reserved word  , which is followed by the same kind of expressions that can appear after  . When *P* appears in the specification of a function, the Move Prover requires that the function aborts if and only if *P* holds. If multiple  conditions are specified, there is an error unless the function aborts if and only if the disjunction of all their conditions holds. (This current semantics of  is subject to change.)

There are two expressions that are specific to the Libra blockchain. The expression  is true iff there is an instance of the type *T* from module *M* appearing under account *A* in the global state tree. In the example of Fig. 4, the first post-condition asserts that the payee account exists after a payment transaction (the payee account might not exist before the payment, in which case it is created). The expression  represents the value of type *T* from module *M* stored at account *A*. In the example, this construct accesses the balance values of the sender (the payer), to make sure that the balance covers the payment, and to assert that the payer account balance has decreased by the payment amount if the payment is successful.

*Specification Translation.* Specifications are translated into  and  statements in Boogie and combined with the prelude (the Boogie model, see Sect. 4) and the translated Move bytecode for the program.

A global Boolean variable  is introduced and assumed to be  at the beginning of each procedure. The Boogie code for each instruction sets this flag to  for conditions that cause abort, such as undefined operations or failures of explicit Move  statements.

The specification translator combines, using logical disjunction, the conditions of all  statements into a single expression (called  here), which is translated into the Boogie specifications  and  .

## Evaluation

In this section, we report on our experience using the Move Prover. We first demonstrate that it can successfully be used on core modules in the Libra codebase.

*Verifying Core Modules.* We wrote specifications for all of the functions (25/25) in the Libra module and most of the functions (34/38) in the LibraAccount module (4 functions use features that are not yet supported: non-linear arithmetic and referencing data in the spec that does not appear in the code).^3^ These are core modules of the Libra system, and their correct execution is crucial. The Move Prover was able to prove all of these specifications in under a minute, as shown below. The modules with their specifications are available in the Move Prover source tree.^4^ The Libra and LibraAccount modules comprise nearly 1300 lines (including specifications). The total size of the generated Boogie files is a little over 14,000 lines, and the generated SMT files are around 52,000 lines. Writing these specifications was quite natural, thanks to the tree-based memory model and to the support for type-generics. Experiments were run on a machine with an Intel Core i9 processor with 8 cores @2.4 GHz and 32 GB RAM, running macOS Catalina.

**Table:** 

Move Module	LoC	Boogie LoC	SMT LoC	Functions	Verified	Runtime
Libra	420	3875	11,688	25	25	2.99 s
LibraAccount	867	10,362	40293	38	34	46.66 s

*Impact of Move Prover.* The Move Prover is co-developed with the Move language itself (which is relatively stable) to ensure that contracts remain correct as the entire toolset evolves. The prover is used in continuous integration, and is beginning to be used to verify contracts in production. As of this writing, the Move Prover hasn’t exposed any serious bugs. However, it has had an impact on how we understand code. An example is a function called  (a version with some specifications and comments omitted appears in Fig. 4). This function simply pays money from the account of the sender (who signed the transaction) to  . In a previous version of the function, the Prover reported errors for two of the “obvious” specification properties shown. The first specification says that the function always aborts when paying zero Libra, because  aborts unless the amount is positive. However, in the earlier version,  handled the payment to deposit the amount in the account when the account did not yet exist, and that payment was allowed to be zero, violating the specification. The function was rewritten as it appears now, so that the same deposit code is called regardless of whether the payee account was newly created. The last specification says that the payer’s account decreases by  after a successful payment. This condition was violated when the payer and payee were the same, resulting in no decrease. Adding an assert (marked “new!” in the figure) to abort in that useless case makes the specification simpler.

## Related Work

The only other formal verification framework for Move that we are aware of is described in [36], where a high-level approach and some case studies are described, but no implementation details are provided.

The closest work in the literature has been done in the context of verification of solidity smart contracts using Boogie. VERISOL [22] is one tool which formally verifies solidity smart contracts via a translation to Boogie. Its specification language is designed for the specific context of application policies, but general specifications can be given by using solidity assertions. SOLC-VERIFY [19, 20] also uses Boogie to perform formal verification for solidity. It includes an annotation-based specification language and supports a larger feature-set of solidity than VERISOL. Interestingly, the formalization of the solidity persistent memory model presented in [20] is similar to our tree-based memory model for Move, though they were developed independently. One novelty of our model in comparison to theirs is its ability to handle generic functions as discussed in Sect. 4 (generics are supported in Move but not in solidity). Both VERISOL and SOLC-VERIFY target contracts written in solidity, and not in the Ethereum bytecode. In contrast, the Move Prover operates on the Move bytecode.

The solidity compiler itself includes a formal verification framework that works via a direct translation to SMT [2]. Several other tools have focused on specific vulnerability patterns, rather than user-defined specifications [16, 28, 34, 40]. Other theoretical foundations have also been employed for the verification of solidity smart contracts. These include the $\mathbb {K}$ framework [35] (see, e.g., [21]), F* [29] (see, e.g., [11, 18]), and proof assistants such as Coq [37] (see, e.g., [42, 43]).

Formal verification of Rust [30] programs is also related to the Move Prover, as Move ’s type system has similar characteristics to Rust [30]. Prusti [4] is a tool that leverages Rust ’s type system information to verify Rust programs. It is based on a higher-level intermediate framework called Viper [33] (that internally uses Boogie in some scenarios). Other verification efforts for Rust employ a translation to LLVM and then leverage LLVM-based verification techniques (see, e.g., [6, 27, 39]).

## Conclusion

In this paper, we introduced the Move Prover, a formal verification tool designed to be an integral part of the process of smart contract development for the Libra platform. Though our initial experience with the Move Prover is positive, there are many avenues for future work that we plan to pursue.

As Move continues to evolve, we expect that some constructs may be easier and more efficient to model by using custom SMT constructs. An example of this is the built-in vector type. Our current model requires the use of quantifiers to compare two vector objects. However, an SMT theory of sequences could be used to model vectors without needing to use quantifiers to define equality. We plan to investigate the use of richer (and possibly custom) SMT theories in our model.

The specifications we have written so far are *local* in the sense that they deal with only a single execution of a single Move function. However, some properties of the Libra blockchain are inherently *global* in nature, such as the fact that the total amount of currency should remain constant. We plan to investigate techniques for creating and checking such global specifications.

The current Prover is still in a prototype phase. But the goal is for it to be a product that is usable by everyone who is writing contracts for the Libra platform. We expect that there will be many challenges in producing a user-friendly, industrial-strength tool, but we also look forward to a future where formal specification and verification is a routine part of the development process for Move modules on the Libra blockchain.

## Footnotes

1 This paper reflects the state of the Move Prover at github commit https://github.com/libra/libra/tree/6798b1cd50ac7d524d3e494783910b3d7e827eef.

2 As with most verification approaches based on generating verification conditions, verifying recursive procedures or loops in Boogie requires writing loop invariants, which can be difficult and may also introduce quantifiers, making the problem harder for the underlying SMT solver. We have avoided this so far by relying on bounded iteration, but our roadmap includes full handling of recursion and loops via loop invariants.

3 Two additional functions in LibraAccount are “native” which means that they are built-in and don’t have any Move code. These are modeled directly in Boogie and are not included in the count here.

4 To reproduce, run  from  in the  source tree.
